# The role of document analysis in health policy analysis studies in low and middle-income countries: Lessons for HPA researchers from a qualitative systematic review

**DOI:** 10.1016/j.hpopen.2020.100024

**Published:** 2020-12-15

**Authors:** Naomi Karen Kayesa, Maylene Shung-King

**Affiliations:** School of Public Health and Family Medicine, Health Policy and Systems Division, University of Cape Town, South Africa

**Keywords:** Document analysis, Document review, Documentary research, Health policy, Policy analysis, Low and middle-income countries

## Abstract

**Introduction:**

Document analysis is commonly used in health policy analysis (HPA) studies, but the purpose and rigour of application is unclear. This review explored the application and utility of document analysis in HPA studies conducted in low-and-middle income countries (LMICs), intending to derive lessons for strengthening this methodology.

**Methods:**

Employing a qualitative systematic review approach, nine electronic databases were searched for LMIC HPA articles that employed document analysis. Articles were subjected to systematic retrieval, storage and quality-assessment. Thematic analysis was used in coding, extraction and analysis of data.

**Results:**

Only 28 studies had sufficiently detailed document analyses and met the inclusion criteria. Document analyses were mainly complimentary to primary data collection forms. The majority, barring four studies, lacked clear purpose and utility in answering the research questions, and rigour in methodology and the reporting thereof. The approach to document analyses bore no relationship to the policy phase investigated. Challenges in accessing documents contributed to methodological difficulties.

**Conclusion:**

Well-executed document analysis has potential to strengthen HPA studies. Health Policy researcher skill in applying this methodology needs strengthening and could be improved by: purposive alignment of the method to research questions; rigorously applying and reporting on search strategy with rigour; source, organize and store documents systematically; apply robust data coding and analysis; and clearly linking document contribution to study findings and conclusions.

## Highlights

• Health policy analysis researchers have always used document analysis.
• The incorporation of documents in health policy analysis processes is unknown.
• Document analysis facilitates validation of data and health policy experiences.
• Well-executed and rigorous document analysis can strengthen HPA studies.

## 1 Introduction

Health policies are critical in shaping and guiding the structure, governance and functioning of a health system. There are various definitions of what constitutes health policy. Contemporary analysts mostly agree that policy, including health policy, takes various forms and while government policy is often considered to be the formally written, official documents, policy is sometimes expressed as unwritten practice, where the practice becomes the policy and constitutes ‘the way things are done’ in an organization or setting [9].

Health policy evolves in different ways and earlier policy analysts somewhat simplistically identified a series of ‘stages’ along a policy journey [34], [62]. Agenda setting, which concerns whether and how a policy issue receives attention, is identified as one of the first stages. Policies then go through a process, often highly contested and spanning several years of ‘development and formulation’, at the end of which, for formal government policies, it goes through a process of legitimation and is then released as official policy. Such policy may be released in various forms and may emerge as a single, or several different documents. For example, government policy on HIV-prevention may be contained in many different documents. Policies are then ‘implemented’ and part of the implementation process involves ongoing ‘monitoring’ and periodic ‘evaluation’ [71].

However, it is now commonly agreed that policy is messy and complex, and whilst different stages may be discernible at different points in the policy journey, these are iterative and emergent, rather than linear and sequential. A sentinel policy framework developed with low-and middle-income (LMICs) country contexts in mind, by [74], emphasizes the important dimensions of *context*, in which policies are developed and implemented, the *process* by which this occurs, the *content* of policies that contain policy intent and prescripts, and the variety of [17] *actors*, comprising individuals, groups and networks, that influence, and are influenced by, policy [50].

Given the length of the policy journey, often several years, the study of health policy through the discipline of health policy analysis (HPA), must take account of the complexity and messiness. Rarely can a single study illuminate all the dimensions and nuances of health policy. Health policy analysis therefore requires that we draw on a variety of disciplines, methods and data sources to answer questions *about* and *for* policy.

Health policy analysis, which forms an integral component of Health Policy and Systems Research (HPSR), concerns the analysis *for* policy, in the development of new or the amendment of existing policy, and analysis *of* policy, meaning the study of how existing policy had come about and/or how policy had been implemented and influenced behaviour and practices in the health system [17], [23], [54]. The World Health Organization (WHO), defines HPA as encompassing ‘decisions, plans, and actions that are undertaken to achieve specific health and health care goals within a society’ [69]. Other analysts define health policy analysis (HPA) as the “multidisciplinary approach to public policy explaining the interaction of institutions, interests and ideas in the policy process” [42]. HPA aids in the understanding of how organizational programs and policies in the health system function and how efficient health systems are in making health services accessible to beneficiaries [54]. Such insights enable learning from past mistakes and making improvements in existing and future policies [16], [39].

Depending on the purpose of the HPA and which dimensions and the phase(s) of the policy journey are examined, the study design, approaches and methodologies differ. Two common approaches to data collection in HPA studies are: primary data collection through, for example key informant interviews and focus group discussions, and secondary data collection, commonly done through document analysis. Documents may be the sole source of data, or complimentary to primary data. As many and varied documents are produced during any one policy journey, document analysis is able to provide significant insights into the what, how and why explorations of HPA studies. Of concern is the lack of diversity in HPA studies, with most adopting primary data collection methods and thus presenting missed opportunities from additional rich data which could be obtained from documentary sources [24].

However, the full range of HPA methods are not always easy to apply in LMICs for various reasons. These range from inadequate resources (both human and material) and political instability among others [2], [33]. Notwithstanding this observation, document analyses are consistently used in HPA studies, including those from LMICs [22]. Among the range of documents employed in research, policy documents, research reports and media reports have been identified as the commonest forms that are incorporated in HPA studies [22], [36].

Despite this widespread use of documents in the social science domains [37], [51], there is no clear framework of how to apply document analyses in HPA studies. The challenges of adopting this methodology or reasons why document analyses are rarely employed as independent data collection methods are not fully understood. More importantly, the way in which these documents are appraised and applied to HPA studies has not been fully investigated.

In this paper we explore, through a qualitative systematic review, the extent to which document analysis is employed in HPA studies and the contribution, methodological and substantive utility derived from employing this method. We posit that document analysis in health policy studies is not given its due consideration, thus missing the potential that this method can offer the health policy researcher. Furthermore, where it is employed, we propose that it is not given the same methodological and analytical rigour, as in the primary data collection components of HPA studies. Through this systematic review we hope to derive lessons and recommendations for health policy researchers when employing document analysis.

## 2 Background

### 2.1 Defining a document and its types

Documents are defined as a range of written material sources available, in relation to a particular topic [27]. They are either produced by an individual for private purposes or an organization or team for public use [65]. Some of the commonly used documents in public policy include media reports, research reports, personal letters, emails, diaries and policy documents (Policy reports, national guidelines and strategies, meeting proceedings, implementation guidelines and training manuals) among others and the use of these may differ depending on the phase of the policy journey. For example the content of position and issue papers, including policy briefs, media outputs and policy round-table deliberations, contain ideas that may help frame policy problems and ideas of how to address these during the agenda setting phase [46], [4], [28], [64]. Different types of documents may be generated in other phases of the policy journey.

While many of the documents associated with a policy journey are public and should be accessible, documents may also be private and not open to the general public (business or non-governmental organization documents) or personal (letters and diaries that are usually not available for public scrutiny). Some, such as minutes of meetings may be difficult to obtain. Accessing and analyzing entire bodies of documents may not be practicable, such as multiple email exchanges between policy stakeholders that may be difficult to extract and analyse [67].

### 2.2 Document analysis

The term ‘document analysis’, synonymously called ‘document review’ is used as a method of accessing data and information in different disciplines and carries different meanings in the way it is conducted, interpreted and applied. As a research data collection method, it is generally described as the systematic collection, documentation, analysis, interpretation and organization of data, printed or electronic [7]. This is used as a sole, or complimentary source of data to answer a research question. Document review and document analysis are often used interchangeably. However, some may regard ‘document review’ as a descriptive and non-analytical process, versus the more empirical and analytical process of ‘document analysis’. In some definitions ‘document analysis’ is considered as a step in a document review, while an opposing view regards document review as the ‘first-pass’ to document analysis. In this paper we will use the term document analysis.

Document analysis involves the process of skimming, thorough reading, examining content and interpretation of documents. Depending on the research question, one may use a rating scale, checklist, as well as a matrix analysis for examining content. Fereday and Muir-Cochrane [21] posit that document analysis involves a focused reading of the document, whereupon the researcher engages in the identification of patterns in the data, and formation of codes and themes on which analysis is based after. Bowen [7] suggests that document review and document analysis both involve the following aspects: (a) the selection and categorizing of relevant documents (b) extraction and analysis of data to draw insights and conclusions about a concept (c) answering of research questions of who, what, where and how, depending on research objectives. Hughes et al asserts that the “*Analysis of data derived from documents is about the search for explanation and understanding in the course of which concepts and theories are likely to be advanced, considered and developed*” [29]. For this reason, data collected from documents must be handled ‘scientifically’ [3]. This implies the application of specified and rigorous processes that are systematically followed to ensure authenticity, representativeness and credibility of data and the ultimate conclusions of the [51]. These systematic processes also involve the identification of irregularities and patterns while collecting data, and paying attention to which data to include and exclude through data condensation, in order to condense large volumes of information [59]. This ensures that every piece of data is reduced without losing its meaning as this would affect how the data is later displayed. Notwithstanding differences in the purpose of studies and the type of documents included, the above are some of the basic guidelines that need to be incorporated when conducting document analysis.

### 2.3 A scoping review of document use in health policy analysis studies

The first step in our study was to conduct a quick scoping of HPA studies that formed part of a database used in a sentinel review by Gilson and Raphaely [25], conducted to examine the extent to which the policy triangle was employed as an analytical framework in HPA studies conducted in LMICs. The review provides a fairly comprehensive data base of HPA studies done in LMICs for the years 1994–2007. Since the release of the Gilson and Raphaely review, several other studies drew on this database to explore methodological questions of interest in HPA [19], [20]. We used the review database to explore the extent to which document analysis was used in the HPA studies and used the insights to inform the questions and methodology of our proposed systematic review.

The scoping review confirmed that document analysis, in one or other form, was employed in most HPA studies conducted in LMICs. Of the 43 articles reviewed, only two used private sources such as diaries and letters as these sources were rarely accessible, and a further two used only media reports. The rest used publicly accessible documents such as policy documents and research reports. Only five (11%) of the 43 studies adopted document analysis as a sole method, whilst the remaining 89% used document analysis as a complimentary method. While this may suggest that document analysis is not robust enough as a stand-alone method, this aspect requires further exploration. Of note was that most studies did not provide adequate detail on how document analysis were conducted and thus called into question the rigour employed for this part of the analysis in these HPA studies.

The studies were also scanned to discern if the stages of the health policy cycle on which the study focused influenced the use of document analysis and the type of documents included. We found no discernible association between the focus of the policy analysis and the use of document analysis. Of the 43 studies, 13 focused on agenda setting, 17 on policy implementation and 13 on a combination of agenda setting and implementation – and in all these studies document analysis was employed as a complimentary data collection method. The utility of the document analysis in understanding the policy experience at a deeper level requires further exploration.

In general, methodological reviews of how HPA studies are conducted in LMICs are sparse. A few recent reviews revealed important insights into the ‘how’ of HPA studies. A review by Erasmus et al. [20] sought to identify the methodological gaps in the way HPA studies are conducted in LMICs and generated thoughts for future analyses. Gilson et al. [26] examined the aspects of discretional power as it relates to policy implementation [26], and the use of street level bureaucracy theory in policy implementation [19] illuminated the use of this method in understanding bottom-up implementation experiences. Walt and Gilson further expanded on a framework to strengthen studies on agenda setting, by exploring how HPA studies in LMICs used an existing analytical framework for agenda setting [66]. Erasmus et al examined how implementation research was conducted in HPA studies done in LMICs. We have not found a similar methodological review on the use of document analysis and this paper, by exploring this aspect, hopes to contribute to the methodological knowledge base for conducting sound HPA studies in the LMIC setting.

## 3 Methods

We employed a qualitative systematic review to explore empirically how document analysis is utilized in HPA studies conducted in LMICs. The initial scoping of the HPA studies in the Gilson and Raphaely [25], provided useful initial insights into the extent and role of document analysis in the HPA studies done in LMICs and helped in shaping the aspects that we set out to explore. As the scoping review revealed that the majority of the HPA studies had used document analysis as either a complimentary or sole method, it gave us confidence that we would find sufficient suitable studies to include in the qualitative systematic review.

Based on the insights gained from the scoping review, we explored the following aspects in this qualitative systematic review: the extent to which document analysis is employed in HPA studies; the purpose for which document analysis was done; the methods used for conducting the document analysis and the rigour with which these were applied and reported on; the range and types of documents used; the utility of the document analysis in understanding the policy experience; how results from the document analysis were used; whether there were any links between the document types and the stage of the policy journey under study. Finally, we explored the facilitatory factors, and the pitfalls and challenges encountered in conducting document analysis in HPA in LMICs in particular, where the material conditions for finding and extracting documents are different to those in HIC where robust archives and databases may exist and be accessible more commonly. These aspects formed the deductive themes that guided our data extraction and analysis of the studies.

### 3.1 The qualitative systematic review process

**Fig. 1:**
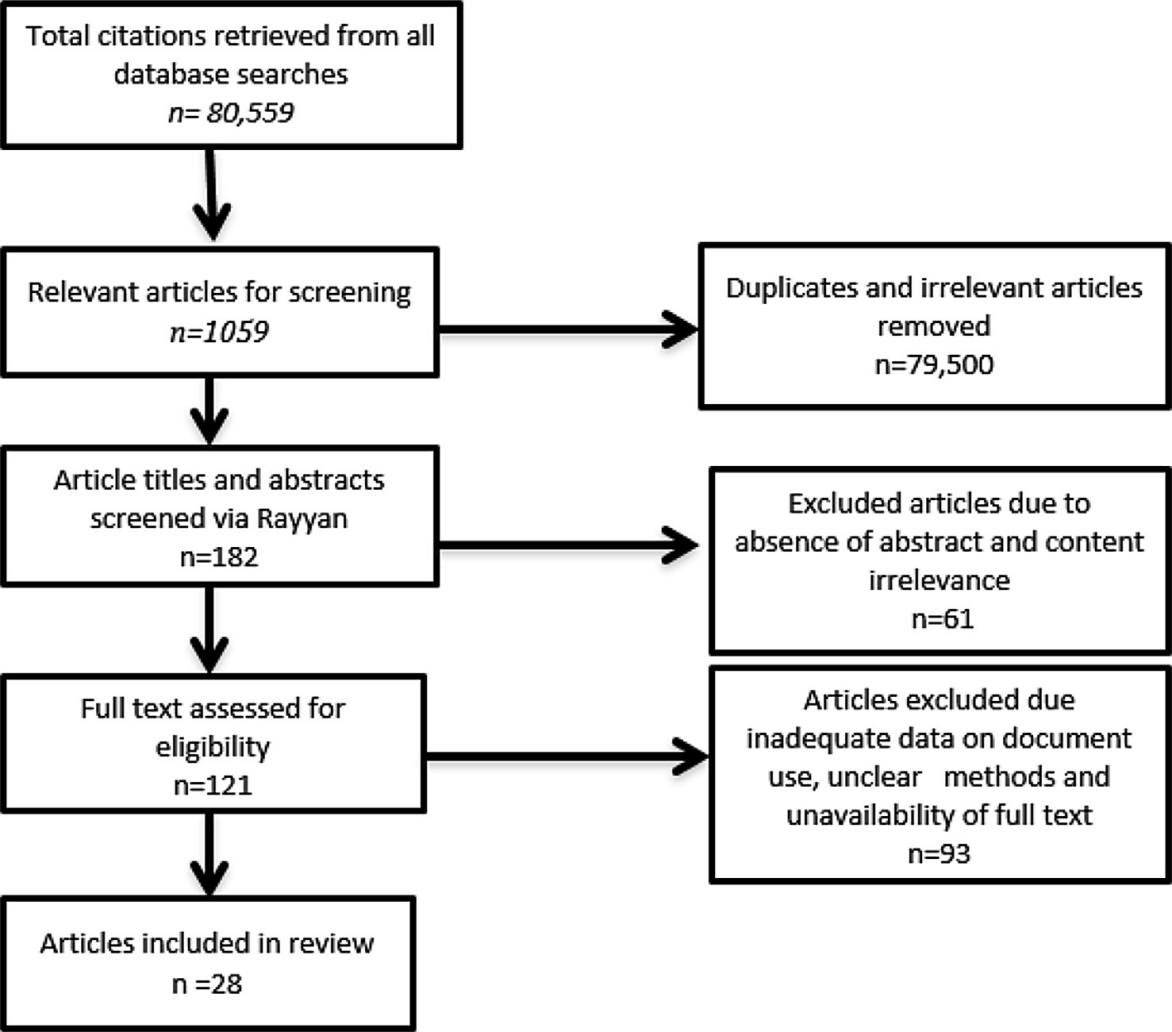
Outlines the step by step article selection process involved in this review.

We followed a Campbell Systematic Review methodology for this qualitative systematic review, to minimize bias in identifying and analysing documents for inclusion and to facilitate the inclusion of a range of HPA studies wherein document analysis was employed as a study methodology. A summary of the review process is presented in Fig. 1.

### 3.2 Search strategy, article selection and quality assessment

Nine electronic databases were searched for relevant articles. The electronic databases included Africa-wide Information, Soc Index, Cumulative Index of Nursing and Allied Health that were searched through EBSCHost, PubMed, Scopus, International Bibliography of Social Science and Web of Science. Keywords and Mesh terms for the review included ‘document review’, ‘documentary research’, ‘health policy’, ‘policy analysis’, ‘document analysis’ and ‘low- and middle-income countries’. All retrieved articles were then transferred to a reference manager EndNote X8 as separate files, identified by their database names. These articles were later merged into a single file, to facilitate the identification of duplicates. All Identified duplicates were then removed automatically by one of the functions in EndNote X8. Upon removal of duplicates, all relevant articles were transferred to Rayyan (available at httt://rayyan.qcri.org), a web application for rapid exploring and filtering of eligible studies in systematic reviews [43].

Based on the inclusion criteria, preliminary sifting through the imported citations in Rayyan was conducted by both authors independently, before decisions were compared. Each author was able to filter, label articles and make comments on why she included or excluded an article, promoting interaction with and tracking of each other’s decision before a unanimous decision was made at every phase (from title and abstract screening to full text reading). This process allowed exhaustive scrutiny, transparency and reproducibility in the article selection. These steps were fully documented and tracked with the use of PRISMA to promote traceability and clarity of the selection process [38].

The articles containing the following characteristics were included at this preliminary phase; HPA studies conducted in LMICs, published in the 2008–2016 period, with document analysis as part of the methodology. Our rationale for choosing the time period beyond the Gilson and Raphaely review was based on the postulate that, as the HPA field has grown in the last decade, the studies done subsequent to 2007 may have become more rigorous methodologically and the lessons that it would provide on the use and application of document analysis may provide more robust lessons for health policy researchers. Studies that adopted qualitative, quantitative, and mixed methods study designs were considered, as long as one of the data collection methods involved document analysis. Only peer-reviewed journal articles with accessible full free text were included, but were limited to those published in English. As we set out to learn about whether, how and to what purpose document analysis was applied in HPA studies, including facilitators and barriers, we only included studies that provided sufficient detail of their document analysis methods and how it was applied in the policy experience. Studies that only mentioned that a document analysis was used, but gave no further detail, were excluded. The exclusion criteria were: all articles published in languages other than English, studies that were: not health policy analyses, conducted in high income countries, published outside the time period, and where the methodology did not include a document analysis.

Following the title and abstract screening, full text reading was done of all articles that met the inclusion criteria. Articles that only mentioned document analysis in their methodology but gave no further detail on how the document analysis was conducted and considered in the findings, discussion and conclusion sections, were then excluded.

The articles that met the inclusion criteria were then assessed for appropriateness and quality using the Critical Appraisal Skills Programme criteria (Public Health Resource Unit: Critical Appraisal Skills Programme [47]. We assessed the article methods, relevance to the area of HPA, overall study rigour, and the adequacy of data on the use and utility of the documents analysis, including the kind of documents used, how they were sourced, how the data analysis was done and whether the data utility was addressed. A scoring system of one to six was developed for scoring the article quality and we only included articles that scored five or six for data extraction. As we ultimately wanted to provide insights on how to conduct a robust document analysis in a HPA study, the quality of the study was of importance.

### 3.3 Data extraction and analysis

We employed a thematic analysis approach [11], [60] in the coding, extraction and analysis of the data. Based on the identified themes of interest as described earlier, a data extraction template was developed in excel, containing the initial deductive themes. Following the coding of the first few articles and extracting the data into the excel template by both authors, the initial deductive themes were refined, the data extraction template modified accordingly, and the coding and data extraction based on the final template, which was then applied to all selected articles.

The descriptive themes included in the data extraction template were: author details, year of publication, study title, study aim and objectives, and study focus in relation to the policy cycle. We then extracted data on: description of documents used (type and number of documents used in the study), sources of documents, facilitators and barriers in identifying and accessing documents, how the document analysis was conducted and used in the study, and the stated contribution of the document analysis to understanding the policy experience (see article characteristics in Table 1). Each article was read line by line, coded as per the final set of themes, and the data extracted into the excel spreadsheet template.

**Table 1:** Article characteristics.

Author(s) and Year of Publication	Country of study	Aim of study	Data collection methods	Focus of study	Findings
4. Abuya et al. [1]	Kenya	To describe the implementation process of the Kenyan output based approach (OBA) program and draw implications for scale up.	Document review and qualitative in-depth interviews	Implementation	Document analysis did not provide all answers about the policy as details around events related to the policy and full views of stakeholders were not found in available reports.
16. Belaid and Ridde [5]	Bukina Faso	To analyse perceptions of policy implementers throughout all stages of the policy implementation process.	Document reviews, interviews and non-participant observations and FGDs.	Implementation	Documents were screened and analysed to better understand the history of the policy and context in which it was implemented.
21. Beran et al. [73]	Multi-country (Kyrgyzstan, Mali, Mozambique, Nicaragua, Vietnam, and Zambia.)	To identify factors that influences the implementation of the policy by policy makers.	In-depth interviews, online questionnaire, and document reviews.	Implementation	Researchers had difficulties accessing documents for the policy analysis and established that failure to access published work hinders the understanding of the impact of policy implementation processes.
22. Bertone et al. [6]	Seirra Leon	To examine the trajectory and determinants of the policy in the post conflict policy environment.	Key informant interviews, stakeholder workshops and document reviews	Policy formulation.	Very little and fragmented documents leading contradictory and vague data found in available documents. However, the few documents found helped formulate preliminary hypotheses and illuminate on gaps from other data collection methods.
28. Chimhutu et al. [10]	Tanzania	To describe the policy process. A qualitative research designs.	In-depth interviews, observations and document reviews.	Policy formulation	Documents helped provide some background information to the study, define questions and trajectories pursued in the other data collection methods. Documents were also helped to uncover the political frames surrounding the policy.
31. Colombini et al. [12]	Nepal	To analyse the historical process of the policy.	Document analysis.	Policy formulation	Despite the provision of background information, documents analysis did not help explain factors leading to contextual and political events leading to the policy.
33. Chimeddamba et al. [76]	Mongolia	To evaluate the extent to which non-communicable diseases (NCD) policies are aligned with WHO NCD control	Document reviews.	Implementation/ policy formulation	Identified that policy processes are not always contained in a document; undocumented policy omissions are also policy actions/inaction. The chronological order of policy documents helped complement existing policy documents and made the policy more popular.
34. Dalglish et al. [13]	Niger	To explore the dimensions of power in health policy making.	Semi-structured interviews, document reviews and contextual analysis.	Implementation/policy formulation	Documents helped with validation of data from respondents, assisted with compiling of the policy’s timeline and political context. However, most were unavailable due to the destruction of WHO-Niger servers by fire in 2007.
40. Doherty [14]	Multicountry (Botswana, South Africa, Uganda, Zambia and Zimbabwe)	To identify major implementation problems with the policy and suggest strategies for better implementation.	Document review and interviews.	Implementation	Found that with incomplete documents, researcher’s meet difficulties in making conclusions about a policy's events leading to its implementation.
44. Doshmangir et al. [15]	Iran	To develop a policy map of the events leading to the milestones of the policy process.	Document reviews and interview	Implementation/ policy formulation	Documents helped clarify different technical terms used by respondents and provided a rich source of information of how the policy entered onto the policy agenda.
45. El-Jardali et al. [18]	Lebanon	To generate insights about how policies are made.	Document reviews and key informant interviews.	Implementation/ policy formulation.	Documents identification was facilitated by interviews and media analysis which helped validate data from interviews and media outputs.
54. Juma et al. [81]	Kenya	To analyse ICCM policy development and the decision-making criteria by policy makers.	Semi-structured interviews, document reviews	Policy formulation	Documents provided the timeline for policy development, policy content and processes. They also informed decisions on the policy and development of training guidelines.
57. Koduah et al. [32]	Ghana	To understand how a policy attained political priority and sustained.	Document analysis, in-depth interviews and participant participation.	Policy formulation	Documents helped map and summarise the historical sequence of events, identifying and classifying policy actors. They also helped triangulate findings from other sources of data.
74. Muga and Jenkins [40]	Kenya	To examine the evolution of the mental health policy from 1965 to 1997.	Document reviews and interviews.	Policy content	Documents helped identify gaps between documented policy progress and actual state of policy by defining the country’s general health policy and distinct historical periods of the current policy.
78. Nguyen et al. [79]	Vietnam	To analyse the medicine pricing policies.	Documentary analysis	Policy formulation	Though documents did not contain answers, they helped identify a reliable and systematic source of data for examining medicine prices applicable to developing countries.
91. Nguyen et al. [77]	Vietnam	To analyse the policy development and understand the obstacles to its implementation	Key informant interviews and document reviews.	Policy formulation and implementation.	Documents provided information on policy content changes, sometimes on the actors, but rarely on how and why these changes happened. Documents helped understand whether changes in one document led to changes in the subsequent policy documents.
92. Place et al. [75]	Mexico	To examine policies regarding postnatal depression	Document reviews.	Policy content	Some policy documents were still in draft form and rendered the HPA inconclusive. A significant number of documents did not contain a specific search term and were excluded leading to loss of documents with potentially useful data about the policy.
94. Rawal et al. [48]	Bangladesh	To aid in the development of appropriate rural retention in Bangladesh.	Interviews, round table discussions and document reviews.	Policy Implementation	Number of included documents increased based on consultation with policy key informants. They identified the need for regular revision of documents as data contained in the documents was old.
81. Odoch et al. [80]	Uganda	To explore the policy process of the introduction of a new policy.	Document reviews	Policy formulation and implementation	Newspaper articles, and other published reports minimized the effects of scarce meeting minutes containing data of the negotiations, formulation and policy implementation.
97. Rodriguez et al. [49]	Malawi	To explore the critical issues in the formulation and implementation of the policy.	Documentary review and in-depth interviews.	Policy formulation and implementation	Documents were used to draw out key events leading to the development of the policy as well as the role and experiences of policy implementers which echoed burn out and unresolved issues related to the policy.
98. Semansky et al. [53]	New Mexico	To examine how the reform impacted the culturally competent services (CCS).	Surveys and document reviews.	Policy Implementation	Documents reviews revealed that for three years the policy was not revised or evaluated to assess the capacity to implement it or evaluate its progress.
100. Singh et al. [57]	South Africa	To determine if oral health elements are coherent with the health policies of post-apartheid era.	Document reviews and interviews.	Policy content.	As there were many different and conflicting documents regarding the same policy, document reviews helped identify one policy document with clear statements on health promotion and oral health and the content therein.
102. Taegtmeyer et al. [58]	Kenya	To examine the policy implications and analyse it against a specific framework.	Document reviews and in-depth interviews.	Policy formulation	Documents reviewed helped identify the absence of data recording with regards to policy’s distinct events and justified why actions were not being implemented by policy makers.
105. Toure et al. [61]	Multicountry (Benin, Chad, Ethiopia, Guinea, Mali, Niger, Swaziland and Togo)	To assess the evolution of African union policies related to women’s and children’s health.	Document review	Policy content and formulation	Found that highly referenced documents and elements acted as entry points for policy issues onto the agenda and sustained issues on the policy agenda. Documents also acted as advocacy instruments as the more articles were written on a policy issue the more attention it got from policy makers.
109. Vuong et al. [63]	Vietnam	To identify the factors that prompted the policy change and its impact on the people using the drug.	Document reviews	Policy formulation	Through document reviews, lack of policy coordination, inconsistencies between legal documents and their contents were identified and acted as evidence to why there was tension between stakeholders and why some policies were being implemented in segregation or failed.
110. Watson-Jones et al. [68]	Tanzania	To explore the feasibility of the policy after introducing to existing policies.	Document reviews and interviews.	Policy formulation and implementation.	Documents were used to verify specific statements and actions in the policies reviewed. Documents reviews identified pertinent issues related to the policy integration onto the larger health services interventions such as financial and human resources limitations.
116. Witter et al. [78]	Sierra Leon	To analyse and document the effects of the free health care Initiatives on health workers.	Document reviews and key informant interviews.	Policy formulation and implementation.	Official documents helped track down the changes to health workers’ incentives in the post-conflict era, set the changes brought by the policy and highlight the current situation and challenges faced by policy implementers.
118. Yothasamut et al. [72]	Thailand	To analyse the process and factors that drove the policy innovation.	In-depth interviews and document reviews.	Policy formulation	Documents helped researchers identify key features of the policy; challenges, cost effectiveness and positions of stakeholders affected by the policy.
118. Yothasamut et al. [72]	Thailand	To analyse the process and factors that drove the policy innovation.	In-depth interviews and document reviews.	Policy formulation	Documents helped researchers identify key features of the policy; challenges, cost effectiveness and positions of stakeholders affected by the policy.

The table provides a summary of the study location, aim for conducting HPA, focus of policy cycle and findings obtained through document analysis.

The thematic analysis of the data involved reading through the extracted text, identifying the key messages for each of the coded themes and presenting it in four overarching analytical themes of: document analysis purpose, document authenticity, document accessibility, methodological rigour applied in the document analysis portion of the study document, the contribution of the document analysis in understanding the policy experience, and the facilitators and barriers experienced in doing the document analysis.

## 4 Results

### 4.1 Article characteristics

A total of 28 articles were included in the final analysis (Fig. 1). Of the 28, two were multi-country studies, which included the following countries; Botswana, South Africa, Uganda, Zambia, Zimbabwe, Mozambique, Nicaragwa, Vietnam, Mali and Kyrgyzstan. Regional location of the studies showed that the majority (16) were done in Africa (Kenya, Bukina Faso, Sierra Leon, Tanzania, Niger, Ghana, Uganda, South Africa and Malawi), nine in Asia (Vietnam, Thailand, Nepal, Mongolia and Bangladesh), two in North America (Mexico and New Mexico) and one from the Middle East (Iran). Of the 28 studies, 24 used a mixed methods approach, in the context of this study meaning that the document analysis was complimentary to other qualitative methods such as focus group discussions, in-depth interviews, round table discussions, key informant- and semi-structured interviews. Four of the remaining studies had document analysis as a stand-alone method.

Across studies the terms ‘document analysis’ and ‘document review’ were used interchangeably to describe similar processes of document identification and selection, data extraction and analyses, and the variety of qualitative methodology techniques to analyse the extracted data.

Despite the widespread use of document analysis as a method, similarly to the studies we explored in the scoping review, only four studies (see Table 2) gave sufficient detail on how the document analysis was done (which included number and types of documents analyzed, selection, quality assessment, data extraction processes, data analysis and learnings from the document analysis). The remainder provided varying degrees of detail on each aspect of the document analysis. Most articles (20) indicated the number and the types (16) of documents analyzed. Only eight indicated the document sources and the selection processes involved. Data extraction was demonstrated in six articles with 12 of them providing details on data analysis approaches, the majority (7 of the 12) of which used thematic analysis. Documents analysis results and learnings were detailed in half of the articles, while the rest presented document-related information intertwined with data collected from other interactive sources and not distinguishing clearly enough between the contribution of the document analysis data and that from other data resources in answering the study questions.

**Table 2:** Approaches to document analysis.

Author(s) and Year of Publication	Aim of study	Focus of study	Description of source, types and quantity of documents given (Y/N)	Selection and quality assessment process done (Y/N)	Systematic data extraction information given (Y/N)	Data analysis processes given (Y/N)	Results and learnings from documents highlighted separately(Y/N)
4. Abuya et al. [1]	To describe the implementation process of the Kenyan output based approach (OBA) program and draw implications for scale up.	Implementation	Yes	No	No	Yes- Thematic analysis and QSR	No
16. Belaid and Ridde [5]	To analyse perceptions of policy implementers throughout all stages of the policy implementation process.	Implementation	Yes	Yes	No	No	No
21. Beran et al. [73]	To identify factors that influences the implementation of the policy by policy makers.	Implementation	Yes	No	No	No	No
22. Bertone et al. [6]	To examine the trajectory and determinants of the policy in the post conflict policy environment.	Policy formulation.	Yes	No	No	No	No
28. Chimhutu et al. [10]	To describe the policy process. A qualitative research designs.	Policy formulation	Yes	No	No	No	No
31. Colombini et al. [12]	To analyse the historical process of the policy.	Policy formulation	Yes	Yes	No	Yes- Thematic analysis	Yes
33. Chimeddamba et al. [76]	To evaluate the extent to which non-communicable diseases (NCD) policies are aligned with WHO NCD control	Implementation/ policy formulation	Yes	Yes- selection process No-quality assessment	Yes	Yes-Thematic analysis	Yes
34. Dalglish et al. [13]	To explore the dimensions of	Implementation/policy formulation	Yes	Yes	No	Yes- NVivo	Yes
40. Doherty [14]	To identify major implementation problems with the policy and suggest strategies for better implementation	Implementation	Yes	No	No	No	No
44. Doshmangir et al. [15]	To develop a policy map of the events leading to the milestones of the policy process.	Implementation/ policy formulation	Yes	No	No	Yes- Thematic analysis	Yes
45. El-Jardali et al. [18]	To generate insights about how policies are made.	Implementation/ policy formulation.	Yes	No	Yes	Yes-Thematic analysis	Yes
54. Juma et al. [81]	To analyse ICCM policy development and the decision-making criteria by policy makers.	Policy formulation	Yes- types of docs No-source	No	No	Thematic analysis	No
57. Koduah et al. [32]	To understand how a policy attained political priority and sustained.	Policy formulation	Yes	Yes	Yes	Yes- Use of pre-existing framework	Yes
74. Muga and Jenkins [40]	To examine the evolution of the mental health policy from 1965 to 1997.	Policy content	Yes	No	Yes	No	Yes
78. Nguyen et al. [79]	To analyse the medicine pricing policies.	Policy formulation	Yes	Yes	No	Yes	Yes
81. Odoch et al. [80]	To explore the policy process of the introduction of a new policy.	Policy formulation and implementation	Yes	Yes	No	Yes	Yes
91. Nguyen et al. [77]	To analyse the policy development and understand the obstacles to its implementation.	Policy formulation and implementation	Yes	No	No	No	No
92. Place et al. [75]	To examine policies regarding postnatal depression	Policy content	Yes	Yes	Yes	Yes	Yes
94. Rawal et al. [48]	To aid in the development of appropriate rural retention in Bangladesh.	Policy Implementation	Yes	Yes	Yes	Yes	No
97. Rodriguez et al. [49]	To explore the critical issues in the formulation and implementation of the policy.	Policy formulation and implementation	No	No	No	No	No
98. Semansky et al. [53]	To examine how the reform impacted the culturally competent services (CCS).	Policy Implementation	Yes	No	No	No	No
100. Singh et al. [57]	To determine if oral health elements are coherent with the health policies of post-apartheid era.	Policy content.	Yes	No	No	No	No
102. Taegtmeyer et al. [58]	To examine the policy implications and analyse it against a specific framework.	Policy formulation	Yes	No	No	No	No
105. Toure et al. [61]	To assess the evolution of African union policies related to women’s and children’s health.	Policy content and formulation	Yes	Yes	No	No	Yes
109. Vuong et al. [63]	To identify the factors that prompted the policy change and its impact on the people using the drug.	Policy formulation	Yes	Yes	No	Yes	Yes
110. Watson-Jones et al. [68]	To explore the feasibility of the policy after introducing to existing policies.	Policy formulation and implementation.	Yes	Yes	Yes	Yes	Yes
116. Witter et al. [43])	To analyse and document the effects of the free health care Initiatives on health workers.	Policy formulation and implementation.	Yes	Yes	No	Yes- Thematic analysis	No
118. Yothasamut et al. [72]	To analyse the process and factors that drove the policy innovation.	Policy formulation	Yes	No	No	Yes- content analysis	No

This table illustrates rigour assessment on documents analysis approaches used in review articles.

Policy documents (which included official government policies, laws, strategies and plans of action, policy guidelines, meeting minutes, policy round-table discussion reports and such) were the main types of documents used in all, but one of the studies. Six out of 28 articles drew on a combination of media reports (mostly newspaper articles), and policy documents). A further six studies used personal letters and diaries and 13 based part of their analyses on research reports that ranged from grey literature to peer-reviewed research papers.

### 4.2 Document authenticity and credibility

According to Abuya et al. [1], documents used in their HPA study lacked detail and did not give a full report of the policy process and views of stakeholders regarding the policy. In instances where a limited number of documents were retrieved, data obtained from the documents were described as fragmented and confusing, vague and sometimes contradictory between document sources [6], [14]. Other authors [57], [63], highlighted that even though documents were relevant to the policy issue, the contradictory information made it hard for analysts to distinguish which source to base their conclusions on. In an example of such contradictions, Singh et al. [57] noted that the aim and guidelines of the oral health policy in South Africa was stated differently in most related policy documents. Clarifying these inconsistencies required detailed engagement with key informants, and whilst triangulating amongst different data sources is important in qualitative studies, it caused undue delays in their data collection process.

### 4.3 Document accessibility

Researchers across studies generally had difficulty in accessing documents, whether as hard copies or on websites (often due to password protection or requiring subscription fees), even when websites and other sources of documents were recommended by stakeholders [41]. This was mentioned in almost a third (10 out of 28) of studies. Some documents were either completely unavailable to the public (in particular private and potentially sensitive documents such as email exchanges), or still in draft form and hence not accessible, destroyed, or simply missing from sites where they were reportedly stored, whether hard copy or electronic. In one example [13], researchers indicated the difficulty with obtaining full details of the events preceding their HPA study, as most of the documents had been destroyed by a fire in one of the World Health Organization (WHO) Niger servers prior to the initiation of their study.

Several researchers bemoaned the fact that failure to access policy-relevant documents limited their understanding of the policy processes, including implementation. In the studies where document analysis was the sole data collection method, researchers reflected that the process of document selection may inadvertently exclude some documents that did not quite meet the inclusion criteria, but may have had some useful information to learn from has also been flagged as an obstruction to document access [48]. While the rigor of document selection is important methodologically, this results in the ‘excluded’ documents becoming ‘inaccessible’ to the study.

Beyond physical access to documents, other barriers were language barriers and incoherent writing and display of information. These were highlighted as inconsistencies in document content and incomplete recording of data, which resulted in gaps for the researchers [57], [58], [63].

Where documents were available, eight studies highlighted the lack of clarity of the data found in the documents, thus questioning the document credibility. Lack of document clarity was attributed to incomplete documents, illegible handwritten data, as well as inconsistent and conflicting information found in documents. Documents written in languages foreign to the researchers inadvertently rendered these inaccessible to them.

One approach used by some HPA researchers for improving the yield from document searches, was to talk to key stakeholders about potential sources of documents [48], [68], [70]. These authors reported that the number of documents included in their HPA studies increased exponentially when they consultation with stakeholders who were directly involved in the policy under scrutiny.

### 4.4 Document analysis purpose and contribution to HPA

We specifically considered whether the stage of the policy journey under study favoured the use of document analysis as a methodology, or favoured the use of particular kinds of documents, but found an unclear association. A number of articles mentioned the key role played by documents and the process of document analysis in aiding their understanding of the four dimensions of policy, as outlined in the Walt and Gilson triangle [10], [11], [12], [49]. Specifically, authors stated that document analysis had helped them better understand the history of the policy process [5], [15], [32], [61], the context in which it was implemented [13], [70], the sequence of policy events [18], [31], [49], as well as the identification of key stakeholders involved in particular policies [63], [72]. It also signalled that the policy triangle was a popular choice of framework for HPA in LMICs. Of note was that, despite the small numbers, where studies focused on understanding the content of policy, document analysis was generally used as a stand-alone method. For most articles that used a mixed method, the purpose of using documents was generally embedded in the overall objective of the study and the purpose of the document analysis was inferred by us, rather than stated explicitly in the study.

Where documents were of acceptable quality, they provided essential insight in a number of ways. Importantly these provided an evaluative base to measure policy actions against stated policy intentions [40], [58], [63]. Documents also aid in historical policy analysis, and for Muga and Jenkins [40] provided insight into the changing policy features across eras, when they studied mental health policy. The shifting tasks of stakeholders’ tasks and the decreasing appropriateness of policy over time, were well-documented and allowed for this temporal analysis. Witter et al. [70] noted greater availability of documents related to their policy of interest during the study period and concluded that it may have reflected rapid activity around the policy at that time, or simply the unavailability of documents for the other time periods that they were interested in. They noted that pre-policy documents were difficult to obtain and resulted in gaps in understanding how historical contexts impacted on the policy they analyzed.

In keeping with qualitative methodology, documents provided an important source for triangulation of data [13], [15], [18], [32] in mixed methods HPA studies, where documents could corroborate or refute the findings, as well as clarify and aid in the interpretation of technical terms, from key informant interviews. It adds to policy knowledge on aspects such as policy challenges, cost effectiveness and stakeholder roles in particular policies [72]. Documents are also described as potential advocacy instruments in instances where they were used to attract attention of policy makers. Toure et al. [61] and Semansky et al. [53] noted that frequently-referenced public documents acted as triggers for getting policy issues onto the agenda. In instances where a policy issue received significant attention in peer-review and popular articles, it got more attention from policy elites.

## 5 Discussion

This study highlights a few key aspects with reference to using document analysis as a method that has an important role and contribution to make in better understanding health policy experiences in HPA studies. To the authors’ knowledge, no other systematic review has been done on the use of document analysis in HPA studies in LMICs. Key insights from this systematic review include the factors associated with selecting document analysis as a method in HPA studies, the potential utility as well as inherent challenges of this method, the methodological gaps and lack of rigour in the application of document analysis by HPA researchers. The lessons from the study may be of value to health policy researchers for designing and executing future HPA studies.

It is undeniable that health policy analysts draw extensively on documents in their studies [44]. However, the majority of studies lacked the methodological rigour required of a document analysis. Whilst some of the studies in the review provided excellent examples of how to conduct robust, rigorous document analysis, the overall body of studies exposed methodological gaps on the part of the HPA researchers in: their understanding of the value of document analysis, in applying rigour to how document analysis are conducted and reported on, and in understanding and exploiting the potential value and contribution of document analysis in understanding a policy experience.

Some of the difficulties with document analysis are inherent to the accessibility and quality of the policy documents, both physical accessibility and accessing good quality information from documents [8]. Here, the nature and type of documents have a strong bearing on accessibility, as some documents that could provide rich insights into a policy experience are simply not publicly available. During the agenda setting phases of policy development for example, and where ideas generation and contestation is at a peak, the documentation of this part of the process is often buried in meeting minutes, email exchanges or ‘private and possibly personal documents’ that are not easy to access [65]. These logistical difficulties in accessing documents dilutes the potential and possibility of using document analysis as a stand-alone method in HPA studies. As health policy contexts are fluid and may not always lend itself to researchers engaging first-hand with the policy environment, document analysis, in LMICs in particular, presents a viable methodological alternative. Ironically the preservation, storing and archiving of documents is also more challenging in these settings.

The challenges with document quality further hamper ‘accessibility’, as poorly constructed documents make it difficult to fully understand and interrogate the dimensions of interest when studying a policy journey or experience. This may be reflective of the capacity of policy makers, where the development and writing of policy documents are not optimal. Several studies highlighted this aspect and alluded to the subsequent dilemmas faced by the policy researcher of having to spend extra resources to verify and validate document content [6], [14], [57], [63]. This may point to opportunities for HPS researchers who are involved in prospective policy analysis especially, as well as for those who regularly interact with policy makers in other spaces of engagement, to provide feedback to policy makers on how to improve the design, content and presentation of policy documents. It will serve both the policy agenda and the researcher agenda, as future studies may then have better document material to work with.

One of the key aspects that we explored in this study was whether document analysis had utility and contributed to understanding the policy experience, whether used as a stand-alone or a mixed method. Whilst the utility is inadvertently influenced by document accessibility and quality, a number of studies highlighted the value-add of documents. Firstly, documents are deemed to be a good source for identifying crucial policy stakeholders and their roles and contribution to the policy process. This aids in any stakeholder mapping and analysis, but also for identification of whom to draw on as key informants. Documents provide the kind of issues that may need further explanation by policy stakeholders, thus serving as a critical source of data in the ‘exploratory’ phase of a mixed-method study. Conversely, it can provide explanations for and corroborate issues raised by interviewees. Where documents provide comprehensive descriptions of policy contexts, processes, and clear policy proposals and intentions, implementation guidelines and implementation experiences, these serve the researcher well in exploring many dimensions of a policy journey and preclude the need for further primary research. This luxury of document-based information is seldom available in full, some policy studies had been able to derive substantial benefit from documents alone. Documents appear to be particularly helpful in historical analysis of a policy experience. As document analysis is also less intrusive and less time-dependent, it allows for a more flexible research timetable [45], [52].

Based on the insights we obtained from the review and drawing on one of the author’s (MSK) experience in conducting policy analysis [30], [35], [55], [56], we venture to offer some recommendations to HPA researchers on how to employ and write up document analysis in HPA studies as outlined in Box 1.

Box 1 **Recommendations for how to conduct a document analysis in an HPA study.**

1. **When choosing to do a document analysis.**

As a first step, the HPA researcher should be aware of the potential usefulness of document analysis as a stand-alone method, or as part of a mixed method study and consider it as a methodology in its own right when conducting a retrospective policy analysis.

The document analysis (review) component of an HPA study must be:

a) purposive, meaning clearly linked to the research question and with a clear intended utility in meeting the study objectives, and.
b) rigorous, meaning all the necessary methodological steps must be followed, documented and described in the study.

Be clear on the assumptions that underlie the expected contributions of the document analysis in understanding the policy issue under investigation.

2. **When doing the document analysis.**

The following steps must be followed and rigorously documented and reported.

*The search and retrieval process.*

Most of the documents used in a policy analysis will be in grey literature data bases (consult librarians if possible) and not conventional peer-review databases and will more commonly require searches of government- and other websites, and sometimes physical searches of offices and archival spaces.

A useful step is to approach key informants who may be able to point out important documents, as well as their locations and who may be able to pave the way to access these for the researcher.

The search process must be documented and reported on rigorously and in detail:

• Stipulate the inclusion and exclusion criteria for the documents.
• Write a detailed account of the inclusion and exclusion criteria, the search strategy, the document identification and extraction process.
• After retrieving the final documents for inclusion, all documents must be recorded, numbered/labelled, and archived in an easily retrievable database accessible to all researchers working on the study. One such database is RAYYAN, that allows for synchronous access to all researchers.

*Data coding and extraction*

Stipulate the intended coding, data extraction and analysis process clearly.

During this part of the process, all documents must be:

• Read thoroughly.
• Coded, using either deductive or inductive approaches. Most HPA researchers use thematic analysis, where the researcher indicates the initial deductive themes and on what basis these were generated. Theories and other empirical studies can be drawn on for the generation of these themes. If an inductive approach is used, stipulate this and if a combination of the two, then stipulate how the addition of the inductive themes altered the analysis.
• Coded text is extracted into a data extraction sheet or codebook. Specify which software package will be used. Some researchers use excel for a small number of documents, others always use NVivo or an equivalent package.

*Data analysis*

The extracted text is now subjected to detailed analysis, keeping the original themes in mind. In making meaning of the masses of text that you may have collected, a few focused analytical themes should emerge, and additional analytical frameworks may be used to make meaning of the data.

*Presenting document analysis findings*

The findings from the document analysis must include:

• The outcome of the search process, usually in a flow diagram.
• A description of the number and type of documents.
• Clear demonstration of how the document analysis contributed to answering the research questions and understanding of the policy experience. As for qualitative studies, where appropriate use quoted text from documents to demonstrate the empirical origin of the analysis.
• If used in a mixed method study, integrate the findings from the document analysis with those from other primary data sources, whilst ensuring the document analysis contribution is discernible.
• Challenges in identifying or accessing documents should be mentioned, to warn future researchers of potential pitfalls in the particular context in which the research was conducted.
• Ethics considerations must be made explicit, especially where private and personal documents are included, where policy stakeholders may subsequently be engaged with on the results and where results may be applied to influence policy and implementation processes.

Box 1 above provides recommendations for document analysis approach in HPA studies.

## 6 Limitations

The authors realize the potential for selection bias, but are confident that this has been minimized as both authors were involved in the screening and selection of the reviewed articles. Additionally, a quality assessment tool adapted from CASP was employed and we used strict inclusion and exclusion criteria. Some of the exclusion criteria were limiting, as only studies published in English were considered. As many of the articles in the review had methodological limitations for the document analysis part of their study, and/or had not fully reported their methodology, our insights derived from the review were thus limited.

## 7 Conclusion

HPA researchers will always draw on documents to understand policy experiences and examine policy processes and their implementation. This review has systematically examined whether and how document analysis is applied and used in HPA studies. Notwithstanding issues of quality and accessibility of documents, it points to inadequate knowledge and understanding in general on the value of this method, how to apply it systematically and with rigour and how to report on it in detail and with rigour. This, suggests that HPA researchers should invest in strengthening this aspect of their capacity, in order to fully exploit the potential of this method. HPA researchers should follow the same systematic and rigorous process in applying and reporting on document analysis as they do for other primary data collection methods, such as conducting interviews for example. Given the constraints that often plague HPA researchers in LMICs in conducting primary data gathering, the use of document analysis, if done well, can contribute significantly to exploring and understanding LMIC policy experiences. The accompanying recommendations provide some guidance for HPA researchers on how to strengthen conducting and reporting of document analysis in an HPA studies.

## Ethics approval

Ethics exemption was granted by the authors’ institute: University of Cape Town, Faculty of Health Science, Human Research Ethics Committee, as the study involved only publicly available and published articles and did not involve engagement with humans as study participants during or after the study completion.

## CRediT authorship contribution statement

**Naomi Karen Kayesa:** Conceptualization, Methodology, Software, Data curation, Writing - original draft, Visualization, Investigation, Writing - review & editing. **Maylene Shung-King:** Supervision, Software, Validation, Writing - review & editing.

## Declaration of Competing Interest

The authors declare that they have no known competing financial interests or personal relationships that could have appeared to influence the work reported in this paper.
